# Impact of the interaction between the polymorphisms and hypermethylation of the *CD36* gene on a new biomarker of type 2 diabetes mellitus: circulating soluble CD36 (sCD36) in Senegalese females

**DOI:** 10.1186/s12920-022-01337-2

**Published:** 2022-08-29

**Authors:** Maïmouna Touré, Abdoulaye Samb, Mbaye Sène, Souleymane Thiam, Cheikh A. B. Mané, Abdou K. Sow, Awa Ba-Diop, Modou O. Kane, Mamadou Sarr, Abdoulaye Ba, Lamine Gueye

**Affiliations:** 1 Laboratoire de Physiologie Humaine et d’Explorations Fonctionnelles, Faculté de Médecine, de Pharmacie et d’Odonto-Stomatologie (FMPOS), de l’Université Cheikh Anta Diop (UCAD), Dakar, Sénégal; 2 Laboratoire de Physiologie Pharmaceutique, Faculté de Médecine, de Pharmacie et d’Odonto-Stomatologie (FMPOS), de l’Université Cheikh Anta Diop (UCAD), Dakar, Sénégal; 3 Laboratoire de Biochimie et de Biologie Moléculaire, Faculté de Médecine, de Pharmacie et d’Odonto-Stomatologie (FMPOS), de l’Université Cheikh Anta Diop (UCAD), Dakar, Sénégal; 4 Departement de Médecine, Université Alioune Diop de Bambey, Diourbel, Sénégal; 5 URL3189 ESS Environnement, Santé, Sociétés, CNRS, CNRST, Bamoko-UCAD, Dakar, Sénégal

**Keywords:** sCD36 protein, Genetic polymorphism, DNA methylation, Type 2 diabetes

## Abstract

**Background:**

Several predisposing factors for diabetes mellitus have been identified, including cluster determinant 36 (CD36) receptor expression. We aimed to determine the effects of *CD36* gene polymorphisms and hypermethylation on the plasma *CD36* protein levels in type 2 diabetes.

**Materials and methods:**

We conducted a cross-sectional study involving 100 females (lean healthy control subjects and subjects with type 2 diabetes). This study was conducted at the Human Physiology Laboratory at the Dakar Faculty of Medicine in Senegal. Circulating sCD36 levels and DNA methyltransferase 3a levels were determined by enzyme-linked immunosorbent assay. The other biological parameters were evaluated in a biochemical laboratory. *CD36* gene polymorphisms and methylation were explored by real-time polymerase chain reaction and methylation-specific polymerase chain reaction, respectively.

**Results:**

sCD36 was negatively correlated with HDL-cholesterol levels (r =  − 0.52 *p* = 0.0001) and triglyceride levels (r =  − 0.36 *p* = 0.01) in control subjects. However, in the type 2 diabetes group, sCD36 levels were positively correlated with total cholesterol levels (r = 0.28 *p* = 0.04). For rs3211867, control subjects harboring the CC genotypes had significantly higher sCD36 levels than control subjects harboring the AA/AC genotype (*p* = 0.02); in the type 2 diabetes group, the sCD36 level was not significantly lower in subjects harboring the AA/AC genotype than in subjects harboring the CC genotype (*p* = 0.27). *CD36* gene methylation reduced the sCD36 level in the control subjects compared to control subjects without *CD36* gene methylation (*p* = 0.03). This difference was not significant in the type 2 diabetes group comparing subjects with diabetes with *CD36* gene methylation to subjects with diabetes without *CD36* gene methylation (*p* = 0.09). We noted a nonsignificant increase in sCD36 levels in subjects with diabetes with *CD36* gene methylation compared to control subjects with *CD36* gene methylation (*p* = 0.27). A combination of the *CD36* polymorphism effect and the *CD36* methylation effect did not significantly reduce sCD36 levels in subjects with type 2 diabetes.

**Conclusion:**

*CD36* gene polymorphisms and *CD36* gene methylation separately reduce sCD36 levels. Their impacts are compensated for in subjects with type 2 diabetes by an increase in sCD36 levels, the mechanism of which needs to be elucidated.

**Supplementary Information:**

The online version contains supplementary material available at 10.1186/s12920-022-01337-2.

## Introduction

Diabetes mellitus is a metabolic disorder that is among the top 10 causes of death in adults, and its incidence is 1.6 times higher in females than in men [1]. From the public health perspective, the identification and evaluation of new biomarkers that could be useful in diagnosing and monitoring type 2 diabetes mellitus (T2DM) before clinical manifestations have been challenging. Thus, it is important to identify biomarkers linked to diabetes mellitus that can be used to further understand its pathophysiology.

Cluster determinant 36 (*CD36*) is a multifunctional signaling molecule with several known ligands, including long-chain fatty acids and both native and atherogenic lipoproteins as oxidized low-density and high-density lipoproteins [2]. The *CD36* gene has been strongly implicated in pathological conditions associated with metabolic dysregulation, such as insulin resistance [3, 4] and type 2 diabetes [5, 6]. Recently, a circulating plasma form of CD36 protein termed soluble CD36 (sCD36) was identified [7, 8]; it is an indirect reflection of *CD36* expression in tissues [9]. Previous studies have indicated that sCD36 is strongly correlated with insulin resistance and the development of type 2 diabetes [7, 8, 10, 11]. Thus, sCD36 is considered a novel biomarker for type 2 diabetes mellitus [7]. Therefore, the expression of CD36 and its reflection sCD36 could be considered an important pillar of this disease. Furthermore, it could explain a large part of the underlying pathophysiology of type 2 diabetes, especially in obese subjects.

Since type 2 diabetes is a heterogeneous disease whose onset and progression depend on genetic and environmental factors, epigenetic mechanisms may also play a key role in the pathology of diabetes and its complications. The evaluation of the effects of genetic and epigenetic variability on the expression of the *CD36* gene and therefore on sCD36 during type 2 diabetes seems interesting. Studies in different populations have reported that several *CD36* variants, including rs1761667 and rs3211867, are associated with changes in *CD36* expression that have consequent abnormalities in fasting glucose and lipid metabolism [12–14]. To the best of our knowledge, there is no research on the associations between sCD36 and *CD36* SNPs and gene methylation in a Senegalese population. Therefore, the objective of this study was to assess the role of sCD36 in type 2 diabetes and the influence of two common *CD36* intronic SNPs, rs1761667 (G/A) and rs3211867 (C/A), and *CD36* gene methylation in Senegalese females.

## Patients and methods

### Patients

This study was conducted at the human physiology laboratory at the Dakar faculty of medicine in Senegal. We enrolled the study population subjects by random sampling. A total of 100 Senegalese females were enrolled in this cross-sectional study (50 healthy control subjects and 50 subjects with type 2 diabetes). The inclusion criteria were an age of 18 years or older for all groups, and subjects were matched according to age. These subjects voluntarily agreed to participate in the study and were selected during the same time period.

For the control subjects, we included females in good health, confirmed by clinical and biological examinations. They were neither pregnant nor breastfeeding. In the survey on the family history of the control subjects, we found that 3 control subjects had at least one family member with a lipid disorder, 6 control subjects had at least one obese family member, and 2 control subjects had at least one family member with type 2 diabetes. The subjects were excluded from the study. This family survey focused only on first-degree relatives (father, mother, brothers, and sisters) (Additional file 1). 

Additionally, we recruited females who were confirmed to have type 2 diabetes and were followed at the national diabetology center of Senegal for their medical follow-up. In this study, type 2 diabetes mellitus was confirmed by clinical and biological examinations (fasting blood glucose and glycosylated hemoglobin levels) according to the World Health Organization (WHO) diabetes diagnostic criteria set in 1979. The exclusion criteria for subjects with diabetes were as follows: pregnant or breastfeeding females and those with other pathological conditions, such as type 1 diabetes mellitus, systemic diseases, inflammatory chronic diseases, autoimmune diseases, tumors, thyroid dysfunction (current hypo- or hyperthyroidism), and liver and kidney diseases (Additional file 2).

The study protocol was carried out according to the Declaration of Helsinki (1989) of the World Medical Association and was approved by our institutional ethics committee of UCAD (Protocole 027512018/CERruCAD). Our experimental protocol conforms to the relevant ethical guidelines for human research. Informed written or oral consent was obtained from all the participants.

### Clinical procedures

At recruitment, all the subjects underwent an interview using a pre-established questionnaire that included demographic characteristics (age, sex, and education level), medical histories (diabetes mellitus and other diseases), and lifestyle habits (smoking, drinking, sports, etc.). The interview was followed by a clinical examination.

Clinical examination and anthropometric measures (weight, height, waist circumference, hip circumference, waist-to-hip ratio, body mass index, and blood pressure) were performed on each subject (Additional files 1 and 2).

### Laboratory biological measurements

Blood samples were obtained the same day in the biochemistry laboratory in the FMPOS of UCAD. Samples were obtained before the interviews after a 12-h overnight fast. Fasting venous blood was collected from all participants at the fold of the elbow of the nondominant arm. For each patient, the collected blood was distributed in a fluoride tube for the determination of fasting blood glucose levels, in a heparin tube for the measurement of lipid levels (total cholesterol, high-density lipoprotein cholesterol (HDL-cholesterol), low-density lipoprotein cholesterol (LDL-cholesterol), and triglycerides) and the renal function evaluation (uremia and creatininemia), and in a tripotassium ethylenediaminetetraacetate acid tube (EDTA K3) for the determination of glycated hemoglobin and DNA extraction. Blood, serum, and plasma were aliquoted and frozen at − 80 °C for further analysis.

Serum glucose concentrations were measured by the glucose oxidase method. Glycosylated hemoglobin was measured by high-performance liquid chromatography. Total serum cholesterol was measured through the reaction of cholesterol esterase/cholesterol oxidase/peroxidase, and total serum triglycerides were measured through the reaction of glycerol-phosphate-oxidase and peroxidase. The LDL-cholesterol concentration was calculated using the following formula: LDL-cholesterol = total cholesterol − HDL-cholesterol − triglycerides/5 [15].

Plasma insulin levels were determined by an enzyme-linked immunosorbent assay (insulin (human) ELISA Kit #A05322.96 wells, version 0118, Bertin bioreagent, France) (Additional file 5).

Insulin resistance was determined by the Homeostasis Model Assessment-Insulin Resistance (HOMA-IR) using the following Matthews formula: HOMA-IR = (insulin (mUI/L) × glucose (mmol)/L)) / 22.5 [16].

Plasma concentrations of human sCD36 were measured using a commercially available CD36 (human) enzyme-linked immunosorbent assay (ELISA) kit (CD36 (human) ELISA kit #KA4204, version 09, abnova, France) (Additional file 6).

Serum DNMT3a levels were measured by using commercially available Human DNMT3a ELISA kits (human DNA (cytosine-5)-methyltransferase 3a, Kit KTE62548, Abbkine, Wuhan, China). In this study, we used the dosage of the enzyme DNMT3a to support the observed methylation of the CD36 gene (Additional file 4).

### Genotyping and methylation analysis

Blood samples for DNA extraction were collected in EDTA K3 tubes. Genomic DNA (gDNA) was extracted from venous peripheral blood leukocytes using the commercially available Spin-column technique kit for DNA extraction from human whole blood (The PureLink® Genomic DNA purification mini kit, Invitrogen™ by Life Technologies, CA K1820-02, lot 1,977,075, Carlsbad, CA 92,008, USA). DNA integrity and concentration were determined by spectrophotometry and electrophoresis. Then, the extracted DNA samples were stored at − 20 °C for future use.

#### Determination of *CD36* gene polymorphisms

The SNP selection for genotyping was conducted following certain conditions. Table 1 shows some of the characteristics of the studied SNPs. To cover a good part of the *CD36* genetic variability in the study, we included 2 tag SNPs (rs3211867 and rs1761667). The criteria used in our SNP selection procedure were as follows: (1) minor allele frequency (MAF) > 0.05; (2) 1 tag SNP block among the 5 large SNP blocks in the HapMap database of 2008: rs3211867 is from block 4 (tagging 8 other SNPs); and (3) 1 tag SNP (rs1761667), which was not present in the HapMap database of 2008 but was chosen based on data from the literature.

**Table 1:** Characteristics of the *CD36* gene SNPs investigated in this study

CD36 variant	Alleles	RefSNP ID	Location
− 31,118	G > A	rs1761667	exon 1A
11,472	C > A	rs3211867	intron 3

The two tag SNPs were detected using standard assays on demand C_8314999_10 and C_1803793_10.

Determination of *CD36* gene polymorphisms was carried out with TaqMan® SNP Genotyping Assays (TaqPath™ ProAmp™ Master Mixes, ThermoFisher Scientific, MA, USA) by the real-time polymerase chain reaction (RT‒PCR) System and allele discrimination technique (TaqMan, Applied Biosystems, Assay Catalog Number 4351379 Foster City, CA, USA) on a 96-well format and read by a StepOne Plus thermocycler (Applied Biosystems, Foster City, CA, USA). The PCR mixture was composed of prepared DNA with distilled water (20 ng in 4.5 μL), 2X TaqMan® Master Mix (5 μL), and working Stock 0X Assay (0.5 μL) to reach a total volume of 10 μL. After an initial step (Pre-PCR Read: Holding Stage) of 30 s at 60 °C and 1 min at 95 °C to activate the AmpliTaq Gold, UP, and enzyme activation, the products were amplified (cycling stage) using 40 cycles of 15 s at 95 °C and 1 min 30 s at 60 °C. Next, the post-PCR read (holding stage) was performed for 30 s at 60 °C. Then, allele detection and genotyping calling were performed using StepOne plus (Corbett Research, Mortlake, New South Wales, Australia) with the available installed software. The amplification parameters were as follows: 95 °C for 2 min; 94 °C for 30 s, 60 °C for 1 min, 72 °C 1 min for 35 cycles, 72 °C for 10 min, and then held at 4 °C until storage using TaqMan SNP assays (Applied Biosystems).

#### Determination of *CD36* gene methylation

##### Sodium bisulfite modification

Genomic DNA was modified with sodium bisulfite using the CpGenome Direct Prep Bisulfite Modification Kit (Catalog No. 17–10,451, Merck KGaA, Darmstadt, Allemagne). Briefly, 500 ng of DNA diluted with RNase-free water (12 µl) was mixed with 13 µl of 2 × extraction buffer and 1 µl of proteinase k, up to 26 µl. The conversion of the bisulfite DNA was carried out in a thermocycler under the following conditions: 8 min at 98 °C, 3 h 30 min at 64 °C, and hold at 4 °C. After conversion and purification, modified DNA was stored at − 20 °C.

##### Methylation-specific PCR (MS-PCR)

**Fig. 1:**
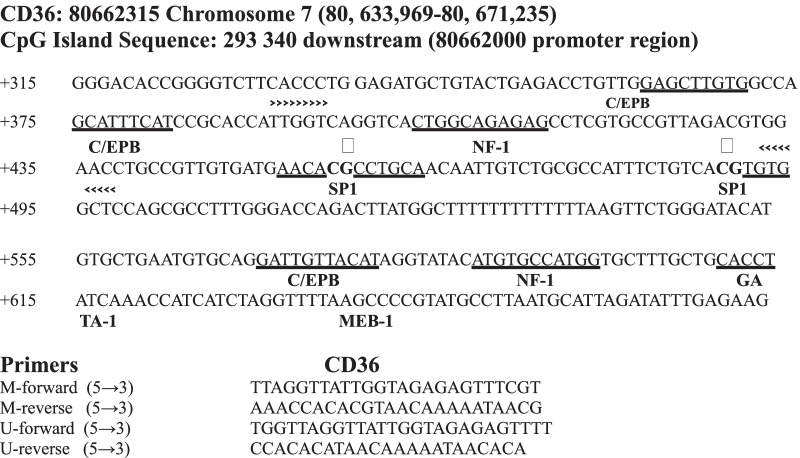
Selection of CpG islands and primer design for methylation-specific PCR (MS-PCR). Human CD36 CpG island sequences (promoter region). Binding sites for transcription factors are underlined. Primer sequences were used for methylation-specific PCR (MS-PCR). Amplified sequences: M-forward primer: primer designed for methylated DNA. U-forward prier: forward primer designed for unmethylated DNA

For the methylation analysis, we selected CpG located at − 293.337 (promoter region) of CD36. Primers for both sequences were designed with MethPrimer. DNA was amplified with two pairs of primers, one for the methylated template and the other for the unmethylated sequence, and the PCR products were 103 bp. Primers for the methylated and unmethylated sequences produced products the same length as the PCR products (Fig. 1) [17] (Additional file 3).

For the PCR assay, 2 µL of bisulfite-modified DNA was amplified in a total volume of 25 µL containing the following: 12.5 µL PCR master mix (Maitre PCR Gotaq* long 2x, Thermo Scientific Inc., USA), 1 µL of each sense and antisense primers, and 8.5 µL of nuclease-free water. For the control, we used human methylated (positive control) and unmethylated (negative control) DNA supplied by the supplier (EpiTect PCR Control DNA Set, Qiagen, USA). For the positive controls, the pretreated DNA showed that the CpG was methylated, and in the same way, in negative control samples, all CpGs were unmethylated. The PCR conditions were as follows: initial denaturation at 95 °C for 4 min; denaturation-extension for 40 cycles at 95 °C for 1 min, 57 °C for 1 min, and 72 °C for 1 min; and a final extension step at 72 °C for 10 min. Amplification was performed in a thermal cycler (iCycler C1000, Bio-Rad, Germany). Finally, 8 µL of PCR product was electrophoresed on a 1% (w/v) agarose gel containing ethidium bromide. The gels were visualized by ultraviolet light (Gel Doc imaging 2000, Bio-Rad).

### Statistical analysis

All variables were saved in an Excel table. Quantitative variables were described using the mean ± standard deviation (SD), and qualitative variables were described using absolute values and percentages. Pairwise comparisons of the study parameters between the control subjects and the patients with type 2 diabetes were evaluated by the unpaired Student’s t test. The chi^2^ test was used to evaluate the association between qualitative variables. The correlation test Pearson was used to assess the associations between sCD36 and the other quantitative variables.

The results were considered significant when *p* ≤ 5%. The data exploitation was carried out by SPSS software version 23.0 (IBM Company, Mexico, Mexico).

## Results

### General and biochemical characteristics

The results of the general and biochemical parameters of the participants according to the different groups are shown in Table 2. We found statistically significant anthropometric and metabolic differences between control subjects and subjects with type 2 diabetes (Table 2).

**Table 2:** Clinical and biochemical characteristics of study participants according to groups

Variables	Control *n* = *50*	T2DM *n* = *50*	*p* value
Mean age (years)	48.98 ± 7.52	50.80 ± 5.93	0.18
Diabetes duration (years)	–	8.89 ± 6.20	–
Waist–hip ratio	0.83 ± 0.09	0.88 ± 0.07	< 0.0001***
Height (cm)	166.50 ± 6.64	164.32 ± 6.75	0.11
Weight (kg)	67.63 ± 9.87	66.26 ± 8.03	0.45
Body mass index (kg/m^2^)	24.25 ± 2.75	24.48 ± 2.33	0.66
Fasting blood glucose (g/l)	0.86 ± 0.13	1.77 ± 0.90	< 0.0001***
Glycated hemoglobin (%)	5.00 ± 0.49	9.32 ± 2.27	< 0.0001***
Insulin (µUI/ml)	17.73 ± 6.37	29.00 ± 15.62	0.01**
IR-HOMA	3.80 ± 1.67	11.82 ± 13.09	0.003**
Total cholesterol (g/l)	2.13 ± 0.44	2.28 ± 0.50	0.12
HDL Cholesterol (g/l)	0.62 ± 0.14	0.56 ± 0.18	0.08
LDL Cholesterol (g/l)	1.40 ± 0.37	1.65 ± 0.53	0.007**
Triglycerides (g/l)	0.80 ± 0.31	0.94 ± 0.55	0.13

*HOMA-IR* Homeostasis Model Assessment-Insulin Resistance, *T2DM* Type 2 Diabetes mellitus, *p ≤ 0.05, **p ≤ 0.01, ***p ≤ 0.0001

### Comparison of the circulating sCD36 protein level according to the groups

**Fig. 2:**
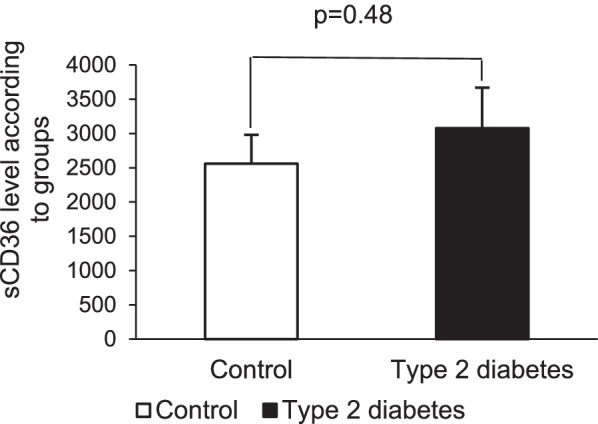
Mean sCD36 levels in the control and type 2 diabetes groups. The symbol without filling is the control group, and the symbol with a single filling is the type 2 diabetes group. A *p* value ≤ 5% was considered significant

In Fig. 2, the sCD36 protein level was not significantly different between the control and type 2 diabetes groups.

### Associations between sCD36 and the other study parameters

Table 3 shows the relationship between sCD36 and the anthropometric, glucidic, and lipidic parameters. sCD36 was negatively correlated with HDL cholesterol (r =  − 0.52 *p* = 0.0001) and triglycerides (r =  − 0.36 *p* = 0.01) in control subjects. On the other hand, in the type 2 diabetes group, sCD36 was positively correlated with total cholesterol (r = 0.28 *p* = 0.04).

**Table 3:** Associations between sCD36 levels and a variety of clinical and biochemical parameters

sCD36 (pg/ml)	Control *n* = 50		Type 2 diabetes mellitus *n* = 50	
	Coefficient	*p* value	Coefficient	*p* value
Body mass index (kg/m^2^)	r = − 0.20	0.15	r = 0.06	0.67
Waist–hip ratio	r = − 0.22	0.12	r = 0.13	0.35
Glycated hemoglobin (%)	r = − 0.20	0.16	r = 0.13	0.36
HOMA-IR	r = − 0.05	0.74	r = − 0.14	0.35
Total cholesterol (g/l)	r = − 0.24	0.09	r = 0.28	0.04*
HDL cholesterol (g/l)	r = − 0.52	0.0001***	r = − 0.07	0.64
LDL cholesterol (g/l)	r = 0.14	0.34	r = 0.24	0.09
Triglycerides (g/l)	r = − 0.36	0.01**	r = 0.09	0.54

*HOMA-IR* Homeostasis Model Assessment-Insulin Resistance, *p ≤ 0.05, **p ≤ 0.01, ***p ≤ 0.0001

### Allelic frequencies and genotypic distribution

Table 4 shows the allelic frequencies and genotype distribution of two *CD36* polymorphisms in control subjects and subjects with type 2 diabetes. The two SNPs were consistent with Hardy–Weinberg equilibrium in each group. We found that the allelic frequencies and genotypic distribution of the *CD36* gene were not significantly different between the two groups (control and type 2 diabetes).

**Table 4:** Association between *CD36* genotypes and the risk of type 2 diabetes mellitus

SNP CD36	Control	T2DM	Statistical calculations Chi^2^; OR; RR; 95% CI
	*n* = 50	*n* = 50	
*rs1761667*			
HWE_X_^2^	0.33 (*p* = 0.57)	0.02 (*p* = 0.88)	*p* = 0.74
VA	0.32	0.29	
Genotyping (%)			
GG	24 (48%)	25 (50%)	Chi^2^ = 0.04; *p* = 0.9
AA + AG	26 (52%)	25 (50%)	
Allele (%)			
A	32 (32%)	29 (29%)	OR = 0.79 [0.43–1.44]
G	68 (68%)	71 (71%)	RR = 0.89; 95% CI
*rs3211867*			
HWE_X_^2^	0.83 (0.36)	0.09 (*p* = 0.77)	*p* = 0.39
VA	0.36	0.36	
Genotyping (%)			
CC	19 (38%)	20 (40%)	Chi^2^ = 0.04; *p* = 0.9
AA + AC	31 (62%)	30 (60%)	
Allele (%)			
A	35 (35%)	36 (36%)	OR = 1 [0.56–1.79]
C	65 (65%)	64 (64%)	RR = 1; 95% CI

*HWE*_*X*_^*2*^ Hardy–Weinberg Equilibrium, *VA* Variation Allelic, *OR* Odds Ratio, *RR* Relative risk, *T2DM* Type 2 Diabetes Mellitus

The study of the genotypic distribution in each group showed that harboring the heterozygous or homozygous genotype of the SNPs considered (rs1761667 or rs3211867) did not lead to a significant difference in the variation in the mean rates of the clinical and biochemical parameters studied (Table 5).

**Table 5:** Baseline population characteristics by genetic variants of the *CD36* gene in each group

Variables	Control			Type 2 Diabetes Mellitus		
	GG	AA/AG	*p* value	GG	AA/AG	*p* value
*rs1761667*						
Waist size (cm)	84.00 ± 10.19	83.54 ± 9.20	0.87	82.32 ± 7.67	86.16 ± 7.30	0.94
Waist–hip ratio	0.82 ± 0.10	0.83 ± 0.07	0.51	0.88 ± 0.07	0.89 ± 0.06	0.64
Body mass index (kg/m^2^)	24.58 ± 2.38	23.95 ± 3.07	0.42	24.30 ± 1.97	24.66 ± 2.50	0.57
Glycated hemoglobin (%)	5.08 ± 0.57	4.93 ± 0.39	0.28	9.35 ± 2.16	9.29 ± 2.42	0.93
IR-HOMA	4.04 ± 1.64	3.57 ± 1.70	0.34	14.04 ± 10.49	9.40 ± 4.55	0.21
Total cholesterol (g/l)	2.32 ± 0.42	2.04 ± 0.45	0.13	2.28 ± 0.42	2.29 ± 0.58	0.93
HDL cholesterol (g/l)	0.63 ± 0.13	0.61 ± 0.15	0.48	0.59 ± 0.17	0.54 ± 0.18	0.38
LDL cholesterol (g/l)	1.46 ± 0.30	1.34 ± 0.41	0.26	1.62 ± 0.47	1.69 ± 0.59	0.63
Triglycerides (g/l)	0.84 ± 0.35	0.76 ± 0.28	0.34	0.95 ± 0.63	0.92 ± 0.47	0.88

	CC	AA/AC	*p* value	CC	AA/AC	*p* value
*rs3211867*						
Waist size (cm)	80.95 ± 9.23	85.48 ± 9.54	0.10	86.90 ± 7.34	85.80 ± 7.55	0.61
Waist–hip ratio	0.80 ± 0.07	0.84 ± 0.09	0.10	0.90 ± 0.08	0.87 ± 0.06	0.25
Body mass index (kg/m^2^)	23.46 ± 2.87	24.74 ± 2.60	0.12	24.46 ± 2.34	24.49 ± 2.20	0.97
Glycated hemoglobin (%)	4.86 ± 0.44	5.09 ± 0.50	0.09	9.77 ± 2.53	9.02 ± 2.07	0.28
IR-HOMA	4.01 ± 1.87	3.67 ± 1.57	0.51	6.41 ± 7.88	9.06 ± 5.02	0.14
Total cholesterol (g/l)	2.11 ± 0.48	2.15 ± 0.43	0.78	2.33 ± 0.60	2.25 ± 0.42	0.65
HDL cholesterol (g/l)	0.59 ± 0.15	0.64 ± 0.13	0.26	0.55 ± 0.13	0.57 ± 0.20	0.61
LDL cholesterol (g/l)	1.45 ± 0.37	1.37 ± 0.37	0.48	1.72 ± 0.66	1.61 ± 0.43	0.49
Triglycerides (g/l)	0.73 ± 0.25	0.84 ± 0.34	0.18	1.07 ± 0.68	0.85 ± 0.44	0.22

*HOMA-IR* Homeostasis Model Assessment-Insulin Resistance

Table 6 divides the entire study population into two groups: a group of subjects harboring the reference homozygous genotype and a group of subjects harboring the heterozygous genotype or the variant homozygous genotype.

**Table 6:** Comparison of baseline population characteristics between groups by genetic variants at  *CD36* gene

	GG			AA/AG		
	Control	T2DM	*p* value	Control	T2DM	*p* value
*rs1761667*						
Waist–hip ratio	0.82 ± 0.10	0.88 ± 0.07	0.02*	0.83 ± 0.07	0.89 ± 0.06	0.008**
Body mass index (kg/m^2^)	24.75 ± 2.61	24.30 ± 1.97	0.50	23.99 ± 3.13	24.66 ± 2.50	0.40
Total cholesterol (g/l)	2.23 ± 0.42	2.28 ± 0.42	0.72	2.04 ± 0.45	2.29 ± 0.58	0.10
HDL cholesterol (g/l)	0.63 ± 0.13	0.59 ± 0.17	0.28	0.61 ± 0.15	0.54 ± 0.18	0.17
LDL cholesterol (g/l)	1.46 ± 0.30	1.62 ± 0.47	0.18	1.34 ± 0.41	1.69 ± 0.59	0.02*
Triglycerides (g/l)	0.84 ± 0.35	0.95 ± 0.63	0.47	0.76 ± 0.28	0.92 ± 0.48	0.13

	CC			AA/AC		
	Control	T2DM	*p* value	Control	T2DM	*p* value
*rs3211867*						
Waist–hip ratio	0.80 ± 0.07	0.90 ± 0.08	< 0.0001***	0.84 ± 0.09	0.87 ± 0.06	0.11
Body mass index (kg/m^2^)	23.46 ± 2.86	24.47 ± 2.34	0.24	24.90 ± 2.80	24.49 ± 2.20	0.52
Total cholesterol (g/l)	2.11 ± 0.48	2.33 ± 0.60	0.22	2.15 ± 0.43	2.25 ± 0.42	0.33
HDL cholesterol (g/l)	0.59 ± 0.15	0.55 ± 0.13	0.39	0.64 ± 0.13	0.57 ± 0.20	0.15
LDL cholesterol (g/l)	1.45 ± 0.37	1.72 ± 0.66	0.11	1.37 ± 0.37	1.61 ± 0.43	0.02*
Triglycerides (g/l)	0.73 ± 0.25	1.07 ± 0.68	0.04*	0.84 ± 0.34	0.85 ± 0.44	0.94

*HOMA-IR* Homeostasis Model Assessment-Insulin Resistance, *T2DM* Type 2 Diabetes Mellitus, *p ≤ 0.05, **p ≤ 0.01, ***p ≤ 0.0001

For rs1761667, the reference homozygous genotype was the GG genotype, and the heterozygous genotype or variant homozygous genotype was the AA/AG genotype. For the rs1761667 SNP, we found that subjects with diabetes harboring the homozygous reference GG genotype had a higher waist-to-hip ratio than control subjects harboring the homozygous reference GG genotype (*p* = 0.02). For the same SNP, rs1761667, subjects with diabetes harboring the AA/AG genotype always had a higher waist-to-hip ratio (*p* = 0.008) and a higher LDL cholesterol level (*p* = 0.02) than control subjects harboring the AA/AG genotype.

For rs3211867, the reference homozygous genotype was the CC genotype, and the heterozygous genotype or variant homozygous genotype was the AA/AC genotype. For this SNP, we found that subjects with diabetes harboring the homozygous reference CC genotype had a higher waist-to-hip ratio (*p* < 0.0001) than control subjects harboring the homozygous reference CC genotype. For the same SNP, 3,211,867, subjects with diabetes harboring the variant AA/AC genotype had a higher LDL cholesterol level than control subjects harboring the variant AA/AG genotype (*p* = 0.02).

### sCD36 levels and *CD36* gene polymorphisms

We found a statistically significant difference in the level of sCD36 only in the control subjects with the rs3211867 polymorphism; the subjects with the CC genotype had a higher mean level of sCD36 than the subjects with the AA/AC genotype (*p* = 0.02) (Table 7).

**Table 7:** sCD36 level variations according to the different polymorphisms

SNP *CD36*	Control	Type 2 Diabetes Mellitus	*p* value
rs1761667			
GG	2145.13 ± 536.30	3415.64 ± 841.03	0.21
AA/AG	2943.04 ± 641.38	2609.80 ± 851.52	0.76
*p* value	0.35	0.50	
rs3211867			
CC	3889.34 ± 776.08	3880.58 ± 1081.11	0.99
AA/AC	1745.31 ± 432.49	2434.15 ± 676.63	0.40
*p* value	0.02*	0.27	

*SNP* Single Nucleotide Polymorphism, *sCD36* soluble CD36, *p ≤ 0.05, **p ≤ 0.01, ***p ≤ 0.0001

### Methylation of CpG islands of the *CD36* promoter

**Fig. 3:**
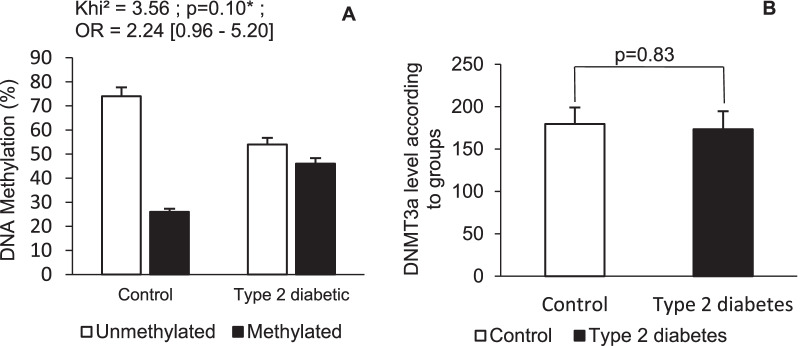
Frequency of *CD36* gene methylation and the DNMT3a enzyme level. In Panel **A**, the symbol without filling is unmethylated, and the symbol with a single filling is methylated. In Panel **B**, the symbol without filling represents the control subjects and a symbol with a single filling represents the type 2 diabetes subjects. A *p* value ≤ 5% was considered significant

Figure 3 shows the frequencies of the methylation of the *CD36* CpG islands in the gene promoter and the DNMT3a level in each group.

*CD36* gene methylation was not significantly different between control subjects and subjects with type 2 diabetes, and females with type 2 diabetes did not demonstrate increased levels of *CD36* gene methylation (Chi^2^ = 3.56; RR = 1.46; OR = 2.24 [0.96–5.21] 95% CI; *p* = 0.10). This result was supported by the fact that the DNMT3a level was not significantly different between the control and type 2 diabetes groups (Fig. 3).

Table 8 shows the parameter variations in the study population according to *CD36* gene methylation. In the control group, subjects without *CD36* gene methylation had a higher triglyceride level than subjects with *CD36* gene methylation (*p* = 0.04).

**Table 8:** Effects of the CD36 gene methylation on clinical and biological parameters

Variables	Control			Type 2 Diabetes Mellitus		
	Unmethylated	Methylated	*p* value	Unmethylated	Methylated	*p* value
Body mass index (kg/m^2^)	24.55 ± 2.84	23.81 ± 3.05	0.54	24.53 ± 2.33	24.41 ± 2.15	0.86
Waist–hip ratio	0.82 ± 0.08	0.83 ± 0.10	0.87	0.88 ± 0.06	0.88 ± 0.07	0.78
Glycated hemoglobin (%)	4.89 ± 0.99	4.55 ± 1.38	0.37	9.05 ± 3.00	9.34 ± 2.06	0.96
IR-HOMA	3.81 ± 1.83	3.19 ± 1.71	0.39	12.89 ± 16.78	9.37 ± 5.22	0.31
Total cholesterol (g/l)	2.09 ± 0.45	2.26 ± 0.41	0.20	2.37 ± 0.58	2.18 ± 0.35	0.15
HDL cholesterol (g/l)	0.61 ± 0.14	0.64 ± 0.13	0.63	0.55 ± 0.13	0.58 ± 0.23	0.52
LDL cholesterol (g/l)	1.36 ± 0.37	1.52 ± 0.34	0.17	1.76 ± 0.58	1.52 ± 0.43	0.10
Triglycerides (g/l)	0.85 ± 0.32	0.66 ± 0.25	0.04*	0.89 ± 0.49	1.00 ± 0.63	0.51

*HOMA-IR* Homeostasis Model Assessment-Insulin Resistance, *p ≤ 0.05, **p ≤ 0.01, ***p ≤ 0.0001

**Fig. 4:**
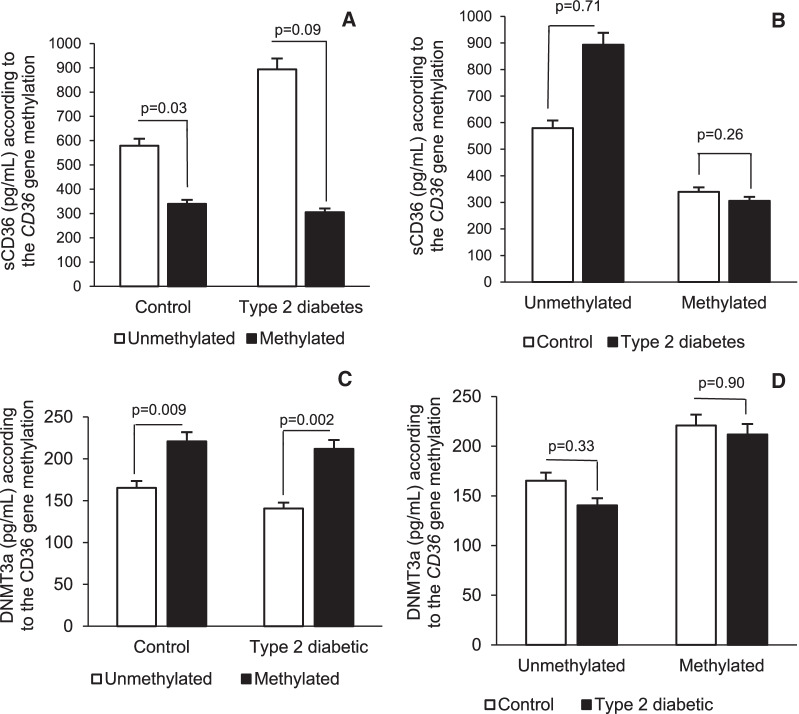
*CD36* gene methylation and its influence on the sCD36 protein level and DNMT3a enzyme level. In Panels **A** and **C**, a symbol without a filling is unmethylated, and a symbol with a single filling is methylated. In Panels **B** and **D**, a symbol without a filling is the control group, and a symbol with a single filling is the type 2 diabetes group. A *p* value ≤ 5% was considered significant

Figure 4 shows the sCD36 protein and DNMT3a variations in the study population according to *CD36* gene methylation and in each group. In each group, subjects without *CD36* gene methylation had a higher sCD36 level than subjects with *CD36* gene methylation. This difference in the sCD36 level was statistically significant in the control subjects (*p* = 0.03). Moreover, the DNMT3a level was significantly increased in subjects with *CD36* gene methylation compared with subjects without *CD36* gene methylation in control subjects (*p* = 0.009) and in subjects with type 2 diabetes (*p* = 0.002).

### Associations between polymorphisms and methylation of the *CD36* gene

**Fig. 5:**
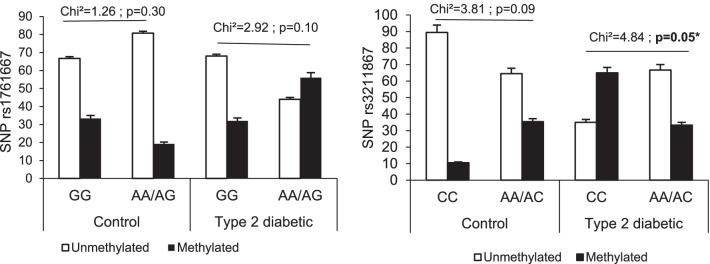
Associations between *CD36* gene polymorphisms and *CD36* gene methylation. The symbol without filling is unmethylated, and the symbol with a single filling is methylated. A *p* value ≤ 5% was considered significant

Figure 5 shows the interactions between *CD36* gene polymorphisms and *CD36* gene methylation in each group.

In the control group, no association was observed between *CD36* gene polymorphisms and *CD36* gene methylation. In the type 2 diabetes group, associations between *CD36* gene polymorphisms and *CD36* gene methylation were noted.

We found that the AA/AG genotype of rs1761667 was significantly associated with *CD36* gene methylation (RR = 2.14; OR = 5.35 [1.59–17.96] 95% CI; *p* < 0.01), and the CC genotype of rs3211867 was significantly associated with *CD36* gene methylation (RR = 2.68; OR = 12.75 [2.29–70.97] 95% IC; *p* < 0.01) (Fig. 5).

### Combined effects of *CD36* gene polymorphisms and methylation on the sCD36 level

In each group, the combined effects of the polymorphisms and methylation of the *CD36* gene on sCD36 levels were not statistically significant, as shown in Table 9.

**Table 9:** Impacts of the combined effects of the *CD36* gene polymorphisms and methylation on the sCD36 level

Variables	Control			Type 2 Diabetes Mellitus		
	Unmethylated	Methylated	*p* value	Unmethylated	Methylated	*p* value
rs1761667						
GG	1609.31 ± 620.30	3216.75 ± 969.51	0.19	2891.94 ± 930.93	4528.50 ± 1763.06	0.43
AA/AG	3340.55 ± 748.39	1273.50 ± 862.93	0.09	1327.27 ± 760.96	3617.50 ± 1364.87	0.16
*p* value	0.08	0.16		0.21	0.69	
rs3211867						
CC	3661.26 ± 846.70	5828.00 ± 1137.50	0.23	2375.06 ± 1167.51	4884.25 ± 1599.21	0.22
AA/AC	1682.95 ± 570.87	1858.68 ± 673.51	0.84	2238.13 ± 793.66	2826.20 ± 1319.28	0.71
*p* value	0.06	0.11		0.92	0.33	

## Discussion

The presence of diabetes can increase a woman’s risk of heart disease twofold. In addition, the presence of diabetes overshadows the protective effects of the premenopausal state [18]. The establishment of markers of insulin resistance and the development of type 2 diabetes has been a challenge and determining point in this field of research. The plasma sCD36 level has been suggested as an adjunct marker for diabetes mellitus [19]. Previous studies have indicated that sCD36 is strongly correlated with insulin resistance and the development of type 2 diabetes [7, 8, 10, 11].

In our present study, the sCD36 level was not significantly different between the control and type 2 diabetes groups (516.72 and 636.95, respectively, *p* = 0.24). The increase in the sCD36 level in the control group was accompanied by a significant decrease in the HDL-cholesterol levels (r =  − 0.52 *p* = 0.0001) and the triglycerides levels (r =  − 0.36 *p* = 0.01). However, in the type 2 diabetes group, an increase in sCD36 levels was associated with a significant increase in total cholesterol levels (r = 0.28 *p* = 0.04).

*CD36* gene polymorphisms were not a risk factor for type 2 diabetes (for rs1761667, OR = 0.79 [0.43–1.44] *p* = 0.9; for rs3211867, OR = 1 [0.56–1.79] *p* = 0.9). A link has been demonstrated between the sCD36 level and the SNP rs3211867. In the control group, subjects harboring the AA/AC genotype had lower plasma sCD36 levels than subjects harboring the CC genotype (*p* = 0.02). This difference in sCD36 levels according to the genotypes of rs3211867 (genotype CC vs AA/AC) was not significant in the group with type 2 diabetes. In addition, we found an increase in sCD36 levels in the subjects with type 2 diabetes harboring the AA/AC genotype compared to the control subjects harboring the AA/AC genotype.

The results show that in the control group, subjects without *CD36* gene methylation had higher plasma sCD36 levels than subjects with *CD36* gene methylation (*p* = 0.03). This difference in sCD36 levels according to *CD36* gene methylation was not significant in the group with type 2 diabetes. In addition, we found a nonsignificant increase in sCD36 levels in the type 2 diabetes group with *CD36* gene methylation compared to the control group with *CD36* gene methylation (*p* = 0.26).

In the type 2 diabetes group, subjects harboring the AA/AG genotype of rs1761667 were fivefold more likely to have *CD36* gene methylation than subjects harboring the GG genotype (OR = 5.35 [1.59–17.96], *p* < 0.01). However, for rs3211867, in the type 2 diabetes group, subjects harboring the CC genotype were 13-fold more likely to have *CD36* gene methylation than subjects harboring the AA/AC genotype (OR = 12.75 [2.29–70.97], *p* < 0.01).

The combination of *CD36* gene polymorphisms with *CD36* gene methylation had no impact on the plasma sCD36 level in the control group or the diabetes group.

The allelic frequencies and genotype distribution of the different *CD36* SNPs (rs1761667 and rs3211867) were not significantly different regardless of the considered group (control or diabetes group). However, previous studies in other populations have shown that *CD36* SNPs are strongly associated with obesity [20–22], which is a major risk factor for type 2 diabetes [23]. The difference between the results obtained here and those of the former studies is most likely due to racial-ethnic discrepancies and several other differences, such as sample size. However, in the control group, subjects with the CC genotype had significantly higher sCD36 levels than those with the AA/AC genotype (*p* = 0.03). These results are in line with those of other authors who have reported other *CD36* SNPs and found that the AA genotype and A allele were characterized by a lower level of CD36 protein expression [22, 24] and a consequent decrease in sCD36 levels. These diminishing effects of the *CD36* polymorphism on sCD36 levels would be compensated for in subjects with type 2 diabetes mellitus. Furthermore, the results showed that in both groups, subjects without *CD36* gene methylation had higher sCD36 levels than subjects with *CD36* gene methylation, and this difference was statistically significant in the control group (*p* = 0.03). This finding corroborates the literature data because DNA methylation has been associated with stable alterations of gene expression and implicated in reducing the circulating level of this protein [25]. However, this condition was not verified in the type 2 diabetes group in this study. The subjects with type 2 diabetes with *CD36* gene methylation had an increase in sCD36 levels and a consequent absence of a significant difference in sCD36 levels compared to subjects with type 2 diabetes without *CD36* gene methylation.

Previous studies have indicated that sCD36 is strongly and positively correlated with insulin resistance and the development of type 2 diabetes [8, 10, 11].

Our results support a potential interaction between genetic variations and DNA methylation in type 2 diabetes. The mechanism linked to this observation remains to be clarified.

We did not observe any particular effect of the combination of *CD36* gene polymorphisms and methylation on the circulating sCD36 level in the control group or the type 2 diabetes group.

Subjects with *CD36* gene methylation had a significant increase in the DNMT3a level in the control group (*p* = 0.009) and in the type 2 diabetes group (*p* = 0.002). We believe that it is quite normal for the DNMT3a level to be higher in subjects with *CD36* methylation. DNA methylation is performed by enzymes from the DNA methyltransferase (DNMT) gene family, the role of which is to affix a methyl group to the DNA cytosines. The DNMT3a enzyme is part of the DNA methyltransferase (DNMT) gene family. It preferentially targets unmethylated DNA and provides de novo methylations [26]. DNMT3a is the enzyme responsible for attaching methyl groups to DNA during replication and de novo methylation [27]. DNMT3a was significantly increased in subjects with *CD36* gene methylation, which further supports their observed methyl status.

sCD36 is involved in macrophage cholesterol and phospholipid transport, and an increase in sCD36 levels reduces circulating plasma cholesterol levels. Studies have already reported an increase in circulating cholesterol by partial or complete *CD36* deficiency [28]. However, we noted an increase in circulating cholesterol levels in subjects with type 2 diabetes despite the increase in the circulating level of sCD36. It would be interesting to elucidate these findings. The CD36 protein has several functions related to fatty acid regulation, such as the transmembrane transportation of LDL [29]. Signal transduction triggered by CD36 ligand binding involves proteins in cellular pathways relevant to some of the metabolic complications of obesity, such as insulin resistance, type 2 diabetes, inflammation status, atherosclerosis, and thrombosis, as previously reviewed [30].

Our study had specific limitations resulting from the use of a small and exclusively female study population. For this, we intend to continue the study on a larger cohort, including males and females, to better establish these results.

## Conclusion

In this study of Senegalese females, the sCD36 level was increased in subjects with type 2 diabetes. sCD36 levels were decreased in subjects with *CD36* gene methylation and in subjects with *CD36* gene polymorphisms (rs3211867). However, these diminishing effects were compensated by the increase in sCD36 levels in subjects with type 2 diabetes. Thus, even a combination of the effects of *CD36* polymorphisms and *CD36* methylation did not significantly reduce sCD36 levels in subjects with type 2 diabetes. We intend to continue the study on a larger cohort to better establish the results.

## Supplementary Information

**Additional file 1**. Sample of the data collection sheet on control subjects.

**Additional file 2**. Sample of the data collection sheet on diabetes subjects.

**Additional file 3**. Corresponds to the gels allowing the determination of the methylated profile or not of the CD36 gene.

**Additional file 4**. Correspond to ELISA plates.

**Additional file 5**. Correspond to ELISA plates.

**Additional file 6**. Correspond to ELISA plates.

## Data Availability

The datasets used and/or analyzed during the current study are available from the corresponding author on reasonable request. Corresponding author email: drmaimounatoure@gmail.com. The ClinVar accession numbers for the submission are: SCV002552556 to NC_000007.14:g.80615623G > A/HP:0,005,978 and SCV002552557 to NC_000007.14:g.80657624C > A/HP:0,005,978.
