# Supplementary material for: Impact of the interaction between the polymorphisms and hypermethylation of the CD36 gene on a new biomarker of type 2 diabetes mellitus: circulating soluble CD36 (sCD36) in Senegalese females
Source: BMC Med Genomics. 2022 Aug 29;15:186. doi: 10.1186/s12920-022-01337-2 (PMC9422098; doi:10.1186/s12920-022-01337-2)
Supplement: Supplementary file 2 — Additional file 2. Sample of the data collection sheet on diabetes subjects. [file 12920_2022_1337_MOESM2_ESM.docx]

**DATA COLLECTION SHEET FOR SUBJECTS WITH TYPE 2 DIABETES ALL FEMALE**

Survey date ………………………………

Patient Index……………………………………. Order number………………………….

Date of collection………………………………… Tel……………………………………….

Address…………………………………………….

**I – Sociodemographic characteristics**

First names………………………………… Last name…………………………

Age:…………. years Ethnicity………………….............

**Occupation :**

Civil servant □ Self-employed □ Volunteer □ Housewife □

Unemployed □ Retired □ Student □ Other □

**Schooling**: Yes □ No □

Level of study language of study…………………………………………………

**II-History and terrain + way of life**

**1-Personal**

** Medical:**

Other illness: no □ yes □

Which one: Hypertension □ Obesity □ Dyslipidemia □

Heart disease □ which one…………………………………………………...

Others: ………………………………………………………………………

** Surgical:**  yes no 

Type of surgery:…………………………………………………………………………..

 **Gyneco-obstetrics**

If woman pregnancy in progress………………………………………………………..

Others:……………………………………………………………………………………..

**2- family history**

Hypertension  Diabetes Obesity  Dyslipidemia 

Heart disease: no  yes  which one…………………………………

Others:………………………………………………………………………….

**3 - Lifestyle**

Smoking: yes □………….. no □

If yes number of packages per day:……….…………………………………..

Duration of smoking:…………………………………………………………

If weaned give date of weaning: ……………………………………………..

Alcohol: yes □………… no □

Sedentary: yes □ no □

Physical activity: yes □…………… no □ Frequency per week:……………………..

**II- Clinical Characteristics**

**1. Constants**

Height:……..m / Weight:………kg / Waist size:………cm / hip circumference:…… cm

BMI:………… kg /m² / Fat mass:…….% / Visceral fat:

SBP:……………… mmHg / DBP:…………….. mmHg / Heart rate:……………. Bpm

**2. Known type 2 diabetes**

Age of onset:……….years Duration of evolution:…………years

Complications: no  yes  which one…………………………………

**3. Treatment**

**Diet**: tracked **** not tracked ****

**Antidiabetics**: biguanides ****  SH **** Glinides **** Insulin **** IAG ****  IDDP-4 ****

**Other associations** :

**Antihypertensives**: ACI ****  ARA2 ****  IC ****  BB ****  Diuretics ****

**Traditherapy**: yes **** no ****

**III- Biological parameters**

In the laboratory

**Lipid profile**

Total cholesterol:………..g/l, HDL cholesterol:…………g/l, LDL cholesterol:…………g/l, Triglycerides :………….g/l

Blood sugar:.…………g/l, HbA1c:………….%, Calcemia:………. g/l

**Prélèvement sang total pour génotypage**
