# Supplementary figures and images for: Impact of the interaction between the polymorphisms and hypermethylation of the CD36 gene on a new biomarker of type 2 diabetes mellitus: circulating soluble CD36 (sCD36) in Senegalese females
Source: BMC Med Genomics. 2022 Aug 29;15:186. doi: 10.1186/s12920-022-01337-2 (PMC9422098; doi:10.1186/s12920-022-01337-2)

# GEL APRES METHYLATION

DIJON

# Gel1: 01 – 07 – 2019

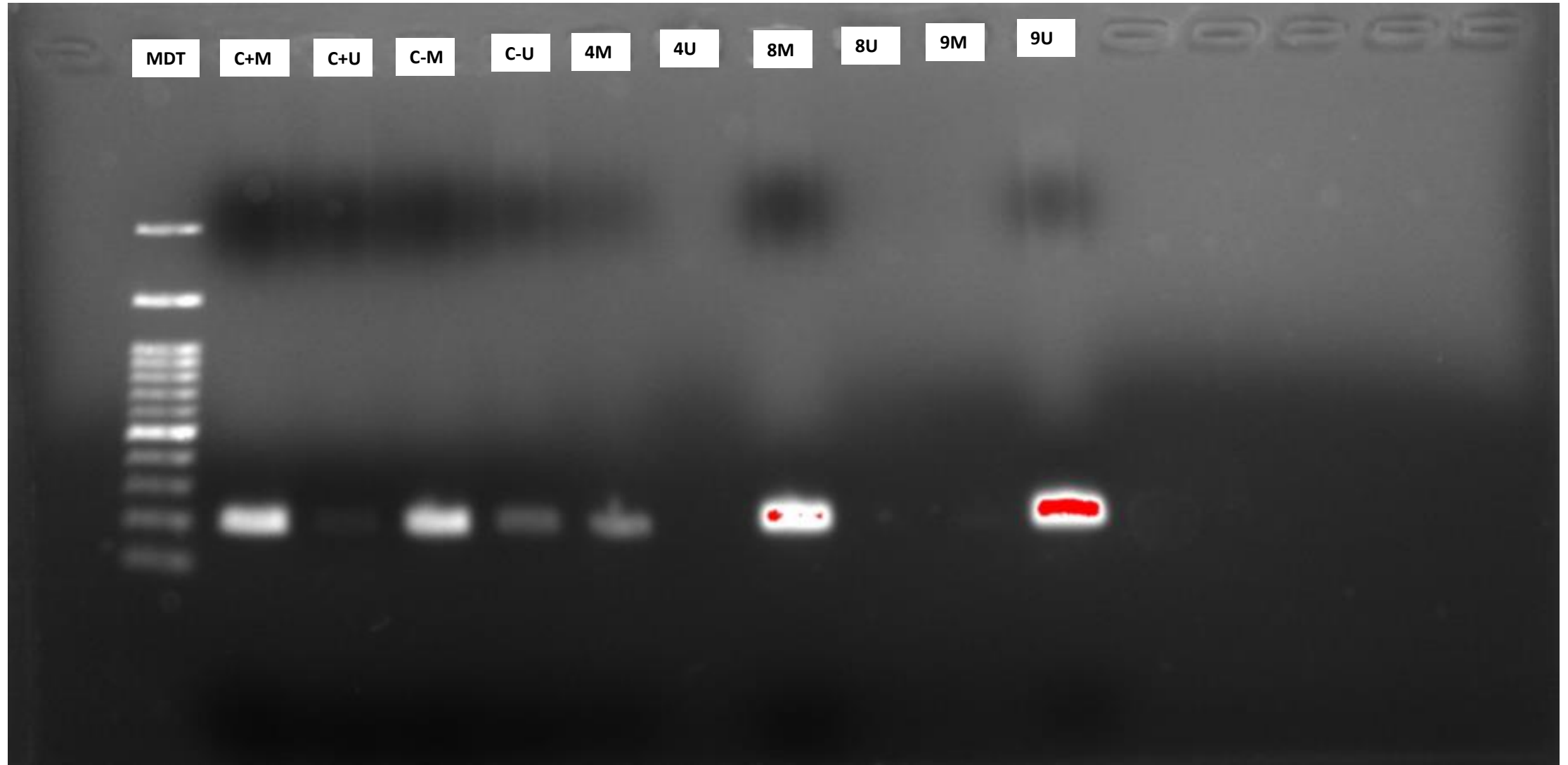

# Gel 2 : 03 - 07 - 2019

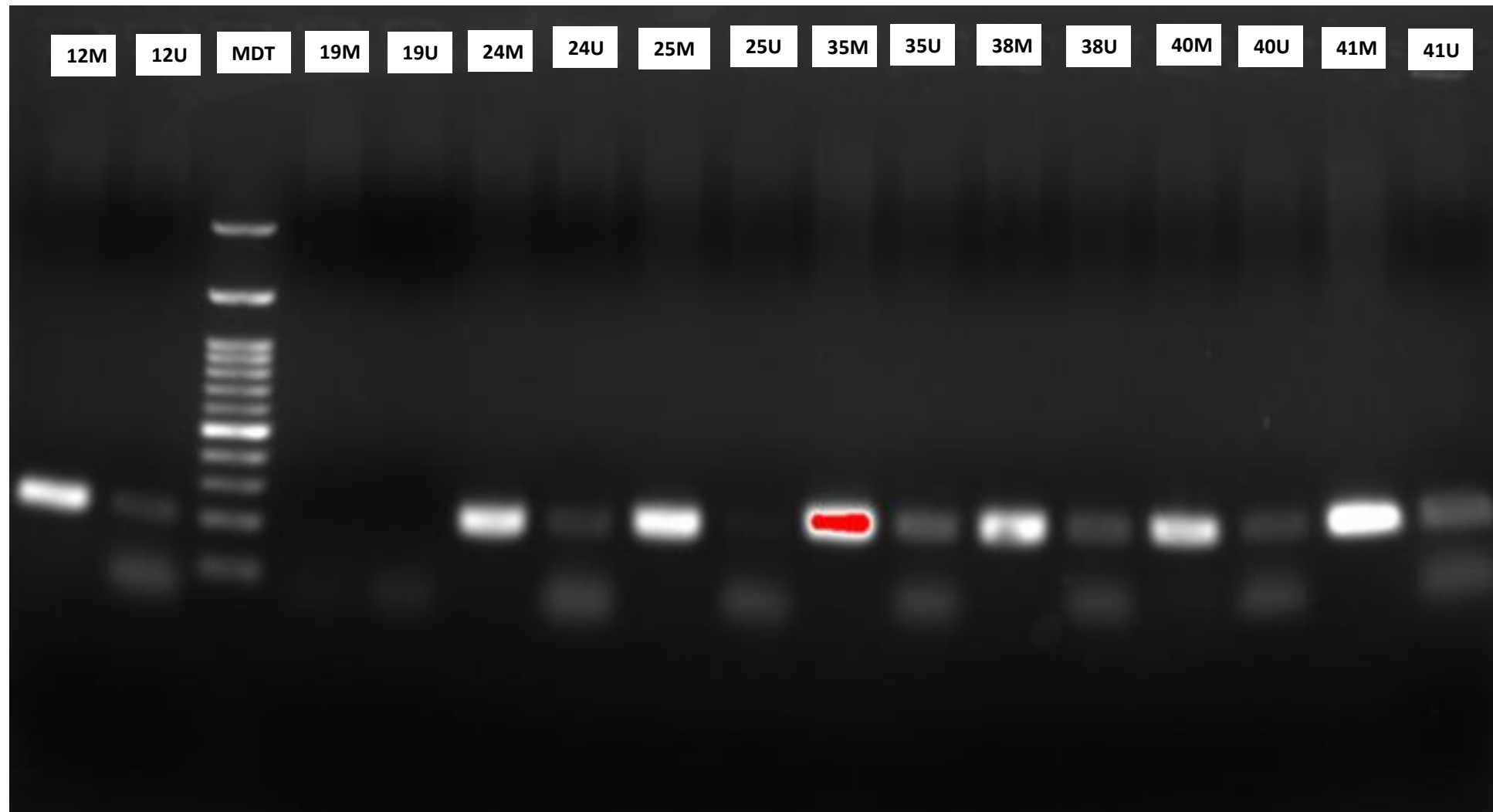

# Gel 3 : 03 - 07 - 2019

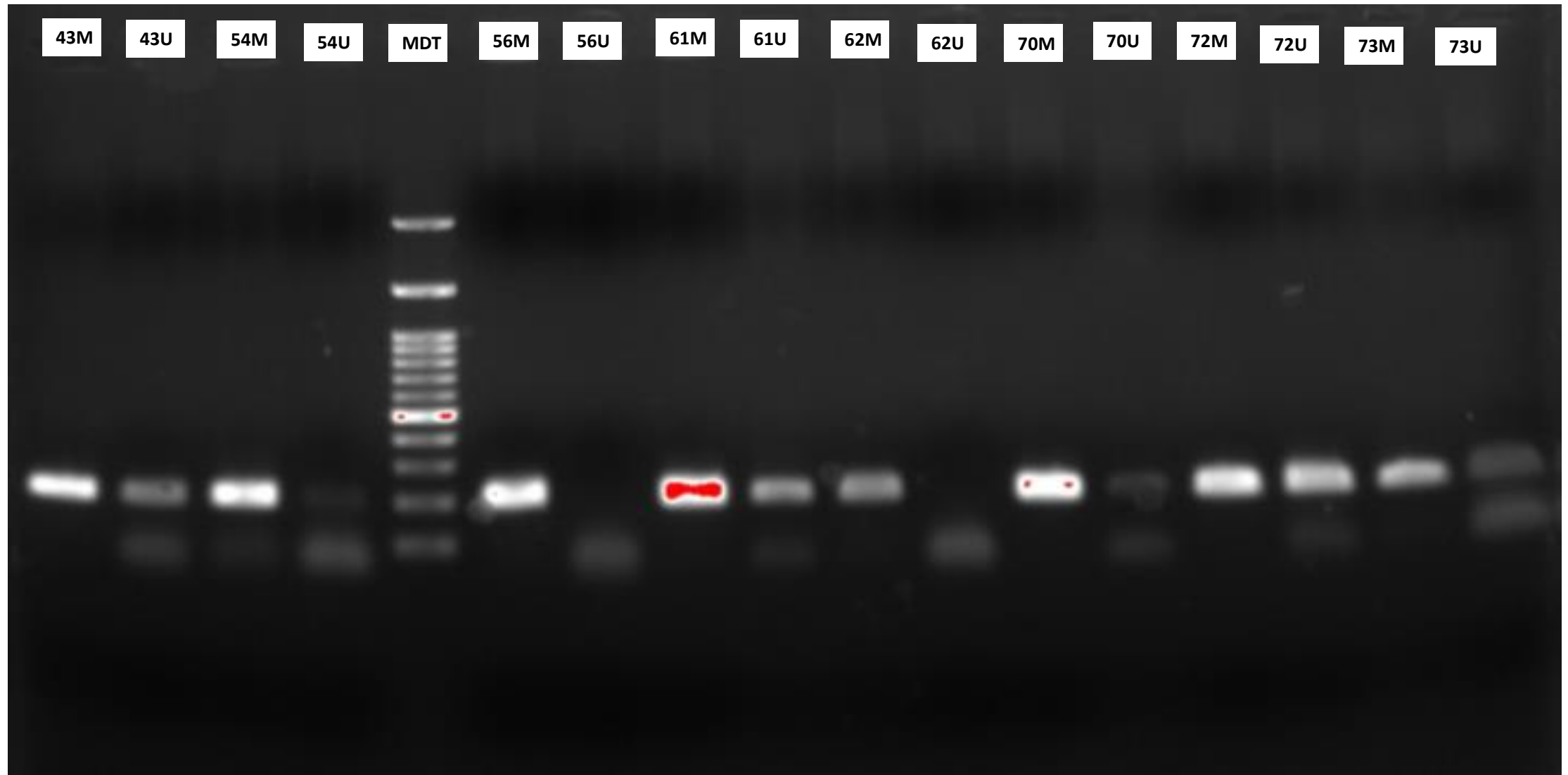

# Gel 5 : 08 - 07 – 2019

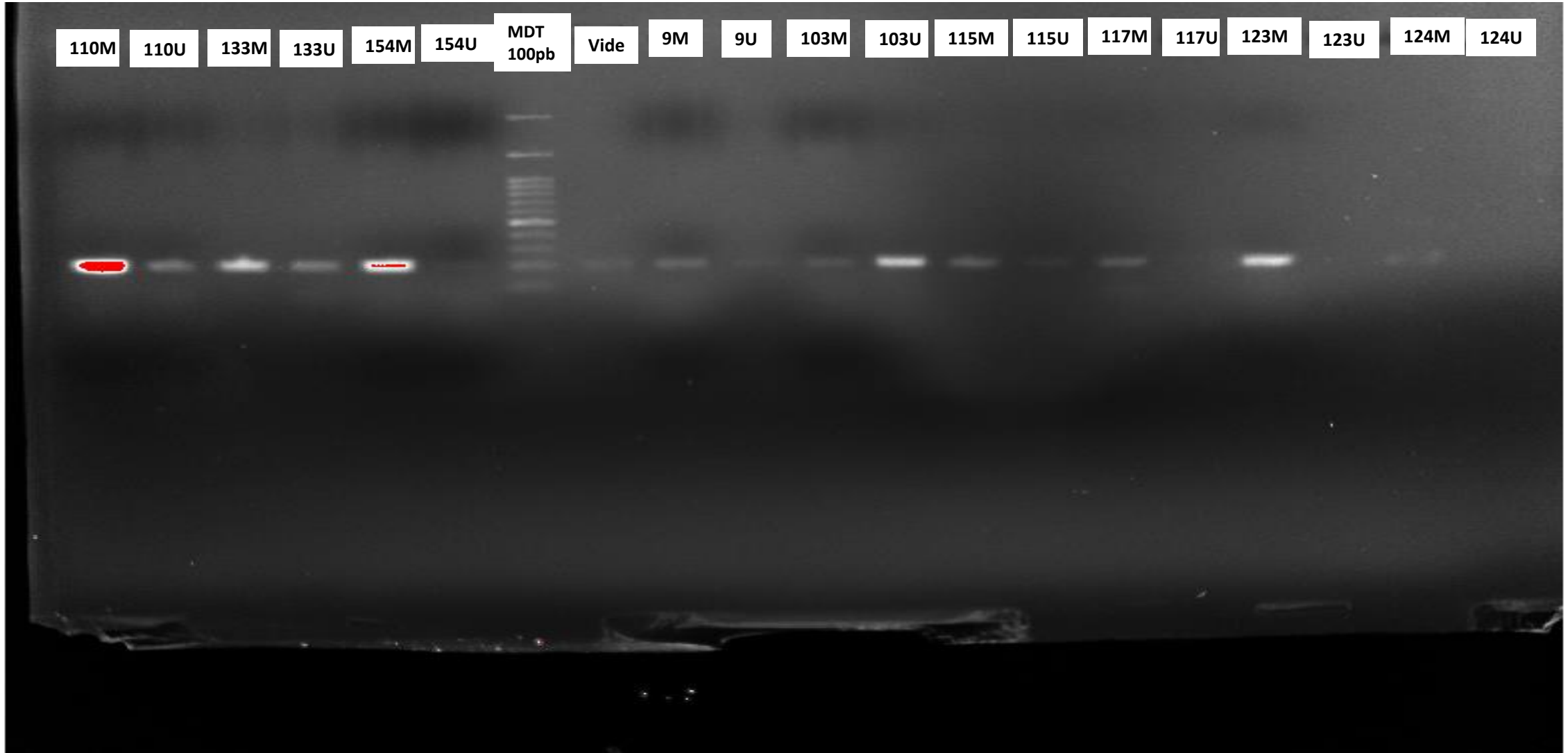

# Gel 6 : 04 - 07 - 2019

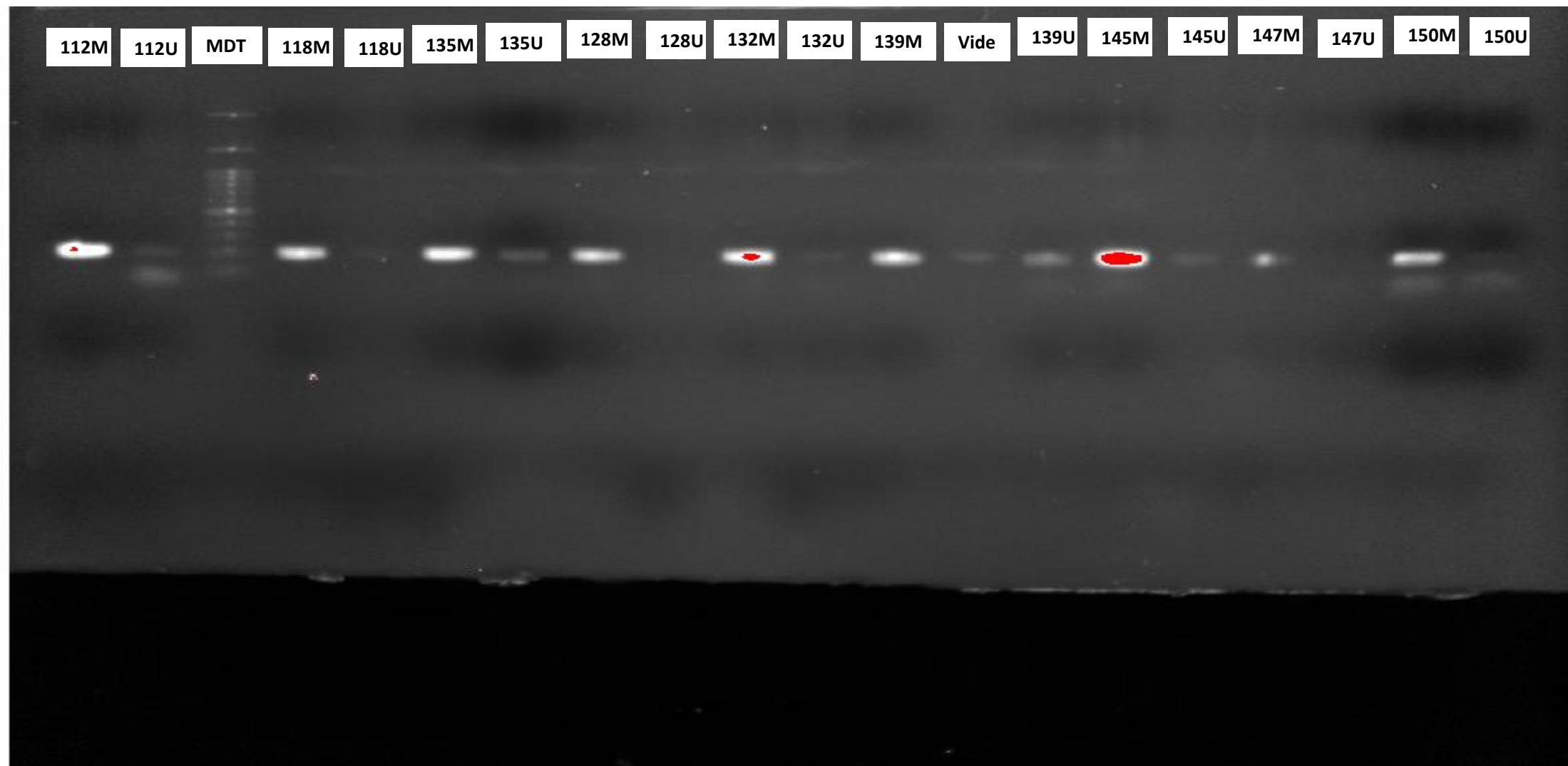

# Gel 8 : 05 - 07 - 2019

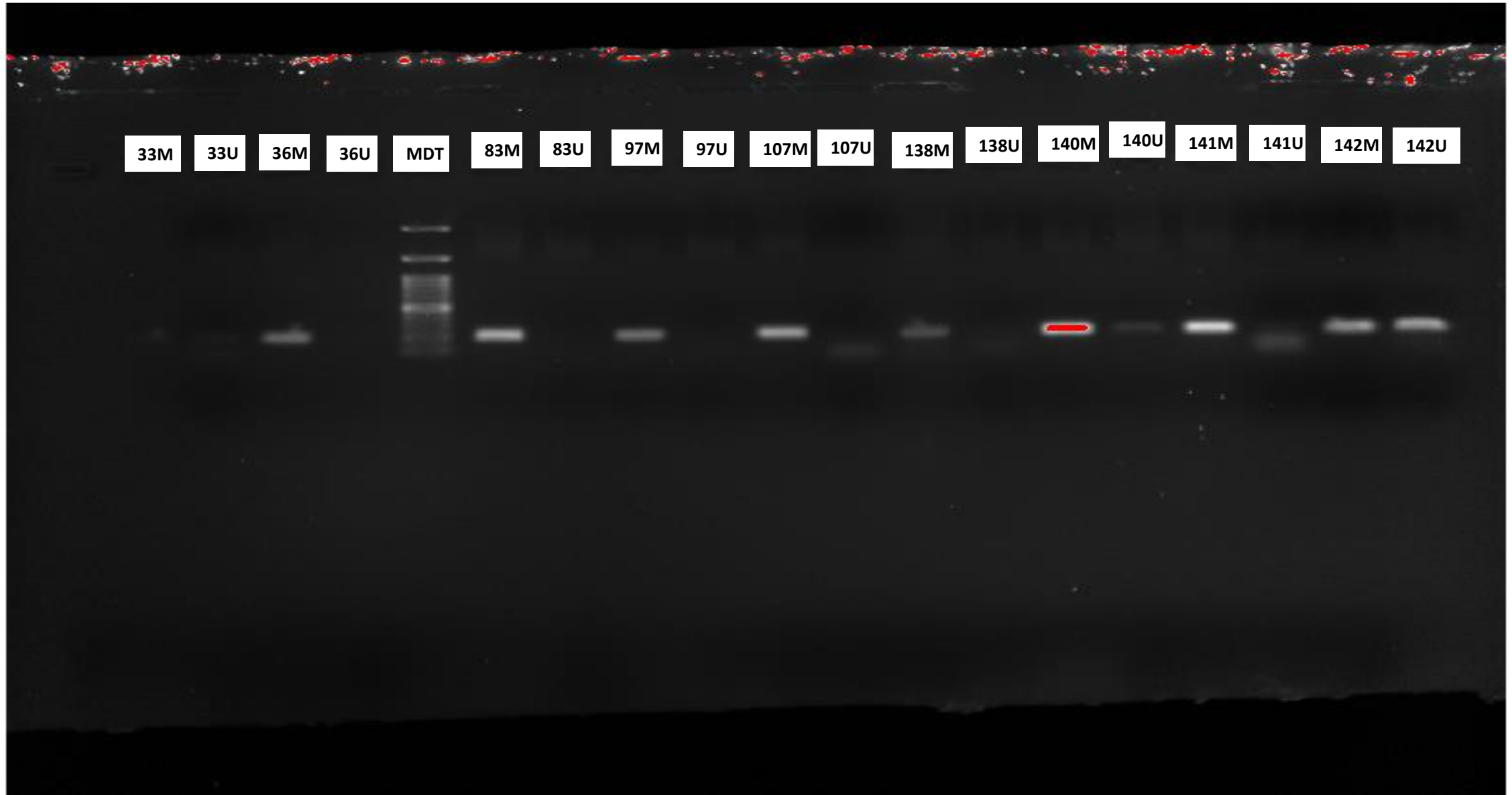

# Gel 9 : 05 - 07 - 2019

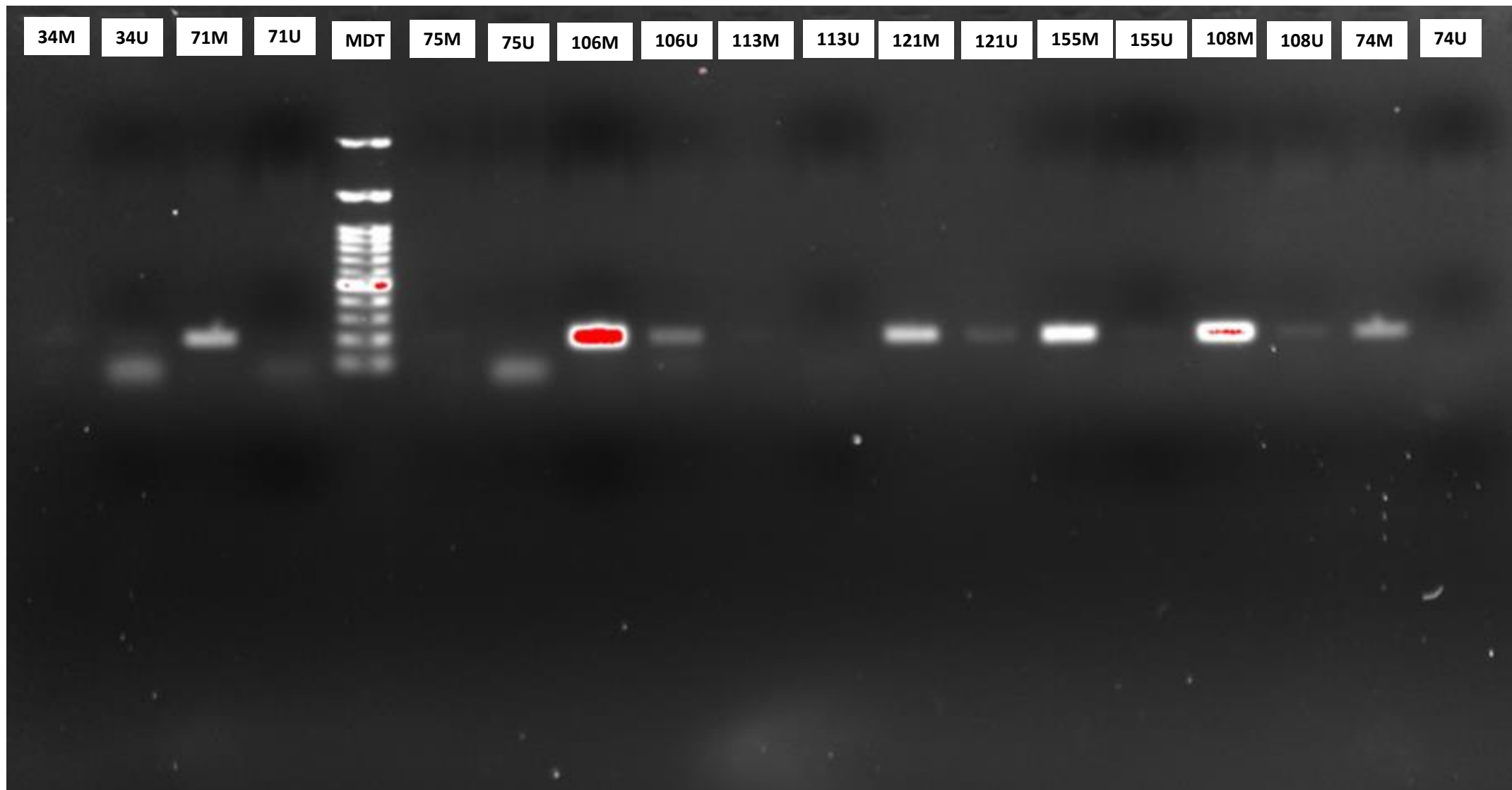

# Gel 10 : 05 - 07 - 2019

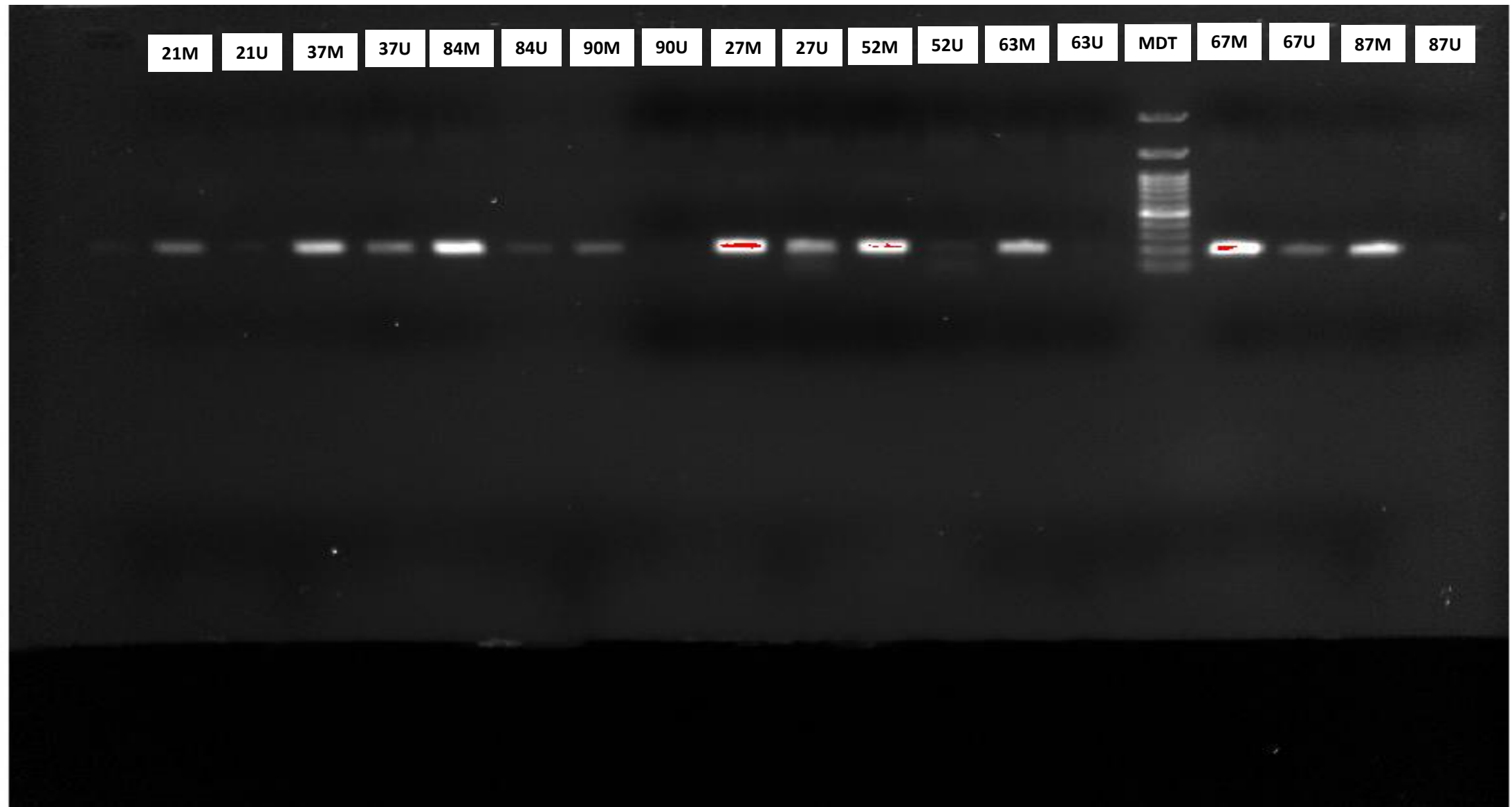

# Gel 11 : 08 - 07 - 2019

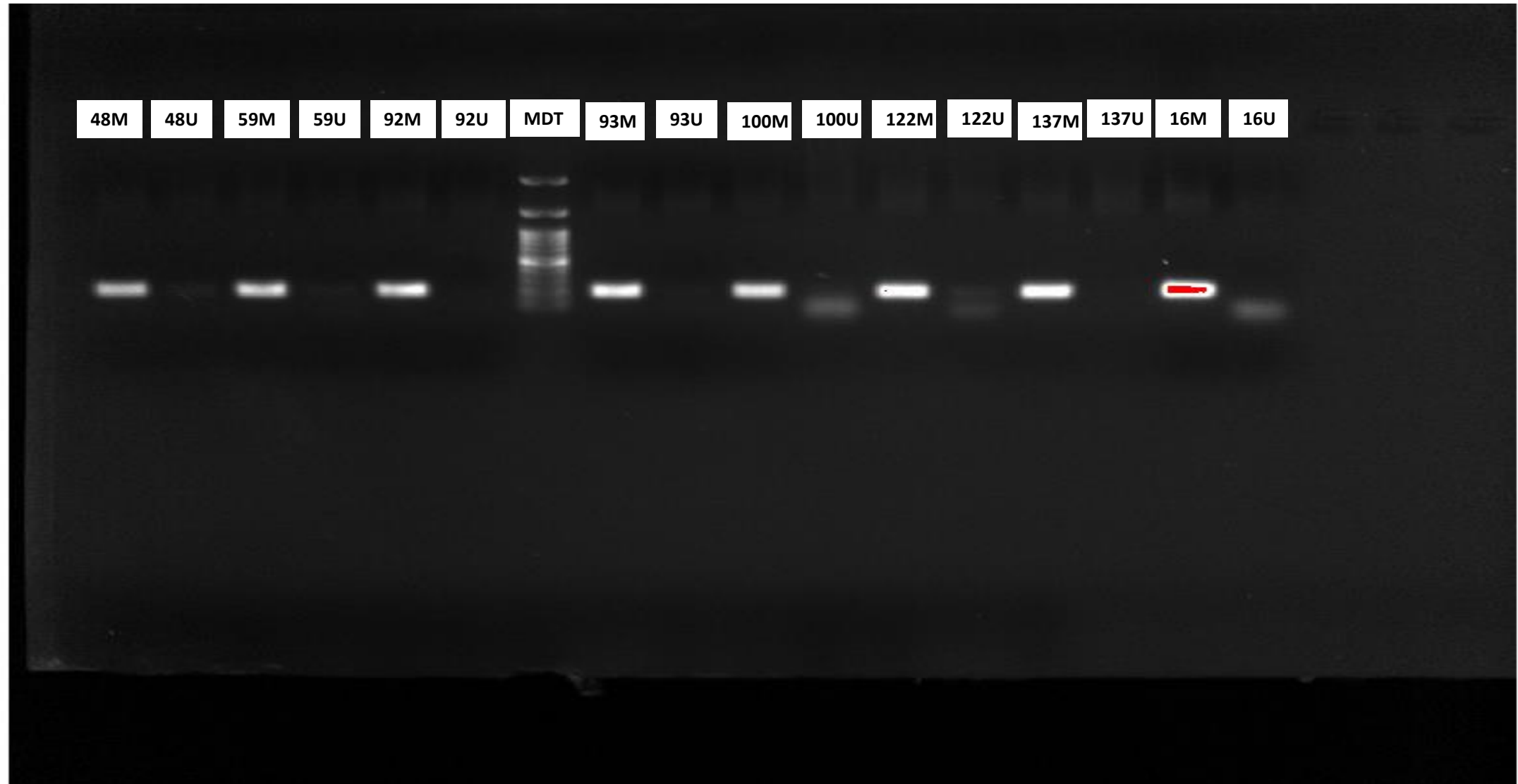

# Gel 12 : 08 - 07 - 2019

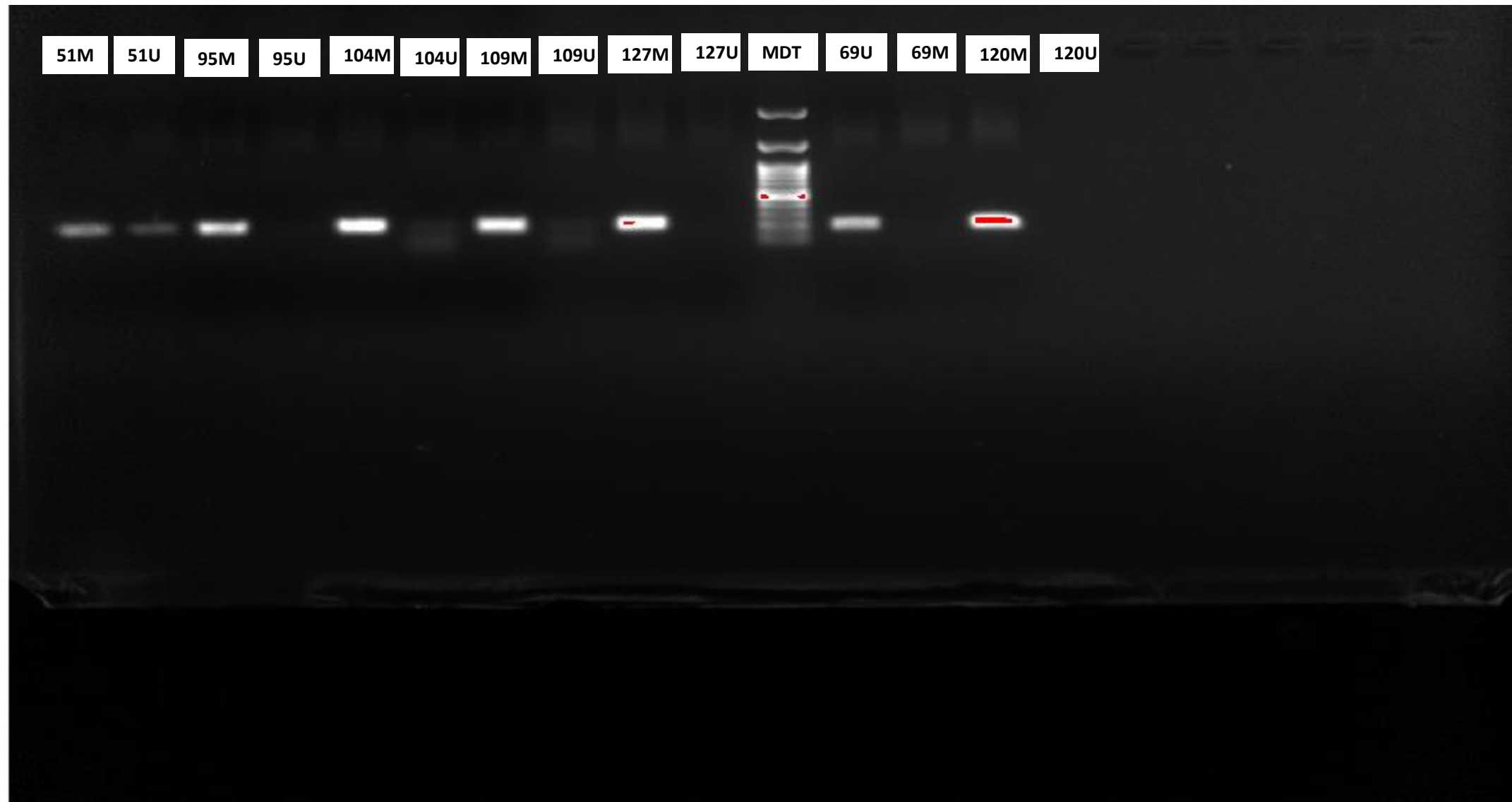

# Gel 15 : 17 - 07 - 2019

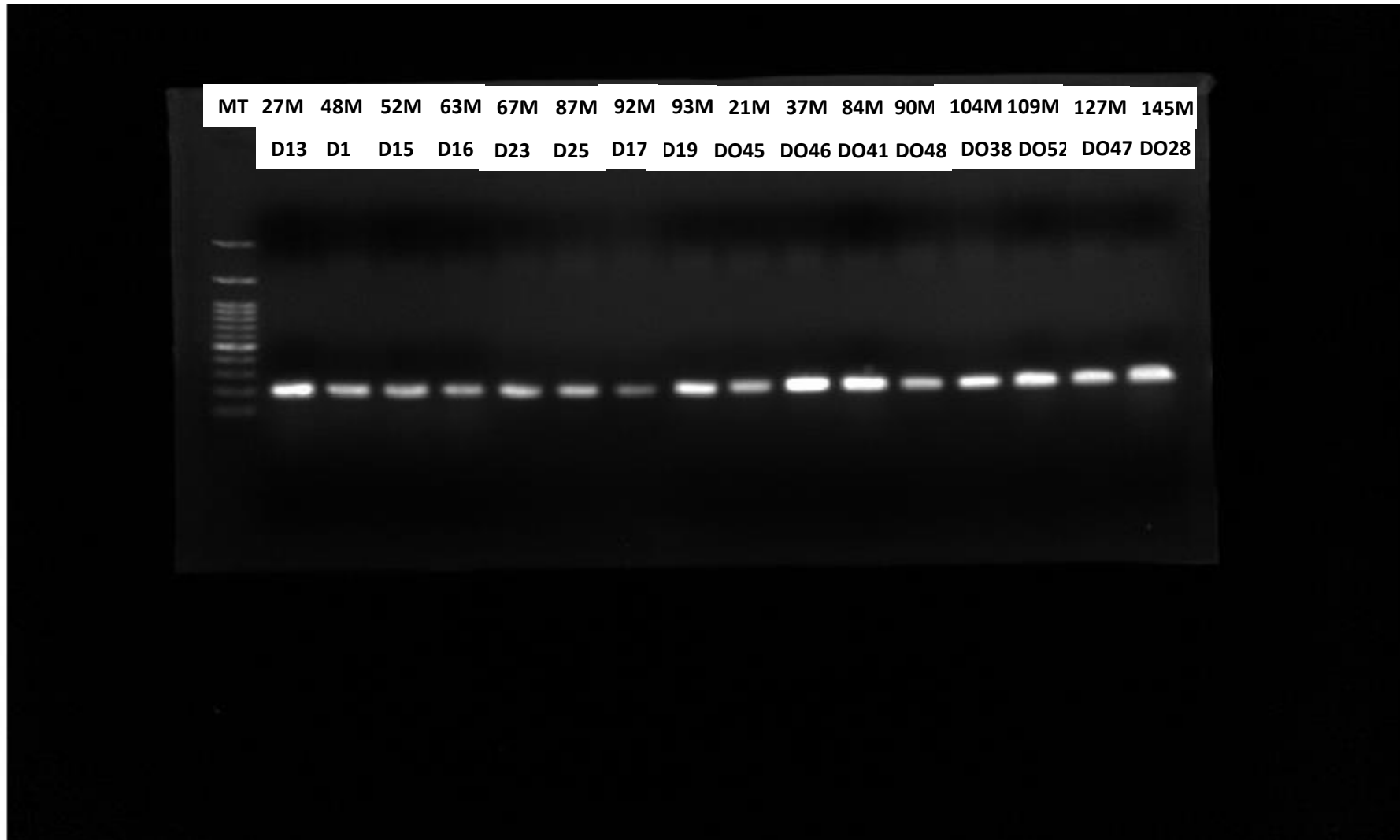

Supplement: Supplementary file 3 — Additional file 3. Corresponds to the gels allowing the determination of the methylated profile or not of the CD36 gene. [file 12920_2022_1337_MOESM3_ESM.pdf]
