# Supplementary material for: Impact of the interaction between the polymorphisms and hypermethylation of the CD36 gene on a new biomarker of type 2 diabetes mellitus: circulating soluble CD36 (sCD36) in Senegalese females
Source: BMC Med Genomics. 2022 Aug 29;15:186. doi: 10.1186/s12920-022-01337-2 (PMC9422098; doi:10.1186/s12920-022-01337-2)
Supplement: Supplementary file 4 — Additional file 4. Correspond to ELISA plates. [file 12920_2022_1337_MOESM4_ESM.pdf]

Human DNMT3A Data by ELISA

8 MIN

Measuremer count: 1 Filter: 450

	1	2	3	4	5	6	7	8	9	10	11	12
A	0,119	0,166	0,153	0,265	0,161	0,151	0,17	0,185	0,225	0,372	0,385	0,307
B	0,153	0,153	0,148	0,206	0,156	0,161	0,15	0,153	0,142	0,19	0,232	0,23
C	0,238	0,238	0,572	0,778	0,142	0,448	0,176	0,139	0,158	0,599	0,233	1,758
D	0,473	0,519	0,132	0,143	0,149	0,141	0,145	0,137	0,21	0,169	0,291	0,755
E	0,72	0,232	0,16	0,23	0,587	0,443	0,206	0,169	0,26	0,239	0,231	0,209
F	1,797	0,199	0,174	0,178	0,169	0,179	0,146	0,184	0,17	0,2	0,205	0,222
G	2,677	0,987	0,194	0,188	0,175	0,199	0,199	0,3	0,257	0,228	0,219	0,305
H	0,25	0,239	0,849	0,163	0,159	0,189	0,184	0,163	0,152	0,184	0,196	0,263

10MIN

Measuremer count: 1 Filter: 450

	1	2	3	4	5	6	7	8	9	10	11	12
A	0,105	0,163	0,152	0,264	0,152	0,147	0,165	0,176	0,222	0,365	0,373	0,299
B	0,132	0,145	0,145	0,198	0,148	0,162	0,151	0,147	0,144	0,191	0,226	0,22
C	0,189	0,226	0,547	0,766	0,136	0,448	0,182	0,132	0,154	0,607	0,229	1,734
D	0,445	0,509	0,126	0,14	0,144	0,145	0,15	0,137	0,207	0,175	0,288	0,743
E	0,678	0,222	0,154	0,221	0,581	0,449	0,211	0,167	0,254	0,245	0,225	0,192
F	1,733	0,191	0,172	0,171	0,166	0,181	0,15	0,183	0,169	0,205	0,199	0,209
G	2,595	0,971	0,185	0,174	0,17	0,204	0,21	0,295	0,25	0,235	0,209	0,294
H	0,224	0,226	0,839	0,155	0,155	0,195	0,19	0,163	0,15	0,19	0,192	0,248
