# Supplementary material for: Impact of the interaction between the polymorphisms and hypermethylation of the CD36 gene on a new biomarker of type 2 diabetes mellitus: circulating soluble CD36 (sCD36) in Senegalese females
Source: BMC Med Genomics. 2022 Aug 29;15:186. doi: 10.1186/s12920-022-01337-2 (PMC9422098; doi:10.1186/s12920-022-01337-2)
Supplement: Supplementary file 5 — Additional file 5. Correspond to ELISA plates. [file 12920_2022_1337_MOESM5_ESM.pdf]

Human insulin data obtained by ELISA

Measurement	count:	1			Filter:	414						
	1	2	3	4	5	6	7	8	9	10	11	12
A	0,124	2,127	0,263	0,185	0,29	0,267	0,2	0,14	0,253	0,562	0,41	0,2
B	0,11	2,562	0,328	0,354	0,19	0,243	0,222	0,139	0,245	0,178	0,22	0,23
C	0,154	0,229	0,392	0,177	0,19	0,266	0,234	0,175	0,239	0,34	0,33	0,14
D	0,202	0,286	0,179	0,292	0,32	0,303	0,157	0,219	0,214	0,39	0,21	0,3
E	0,322	0,544	0,171	0,181	0,43	0,371	0,261	0,232	0,151	0,562	0,22	0,19
F	0,514	0,166	0,378	0,248	0,32	0,183	0,225	0,208	0,248	0,223	0,25	0,2
G	0,839	0,296	0,366	0,167	0,42	0,301	0,183	0,272	0,215	0,411	0,22	0,3
H	1,422	0,124	0,243	0,181	0,38	0,277	0,193	0,17	0,154	0,491	0,38	0,37
