# Supplementary material for: Impact of the interaction between the polymorphisms and hypermethylation of the CD36 gene on a new biomarker of type 2 diabetes mellitus: circulating soluble CD36 (sCD36) in Senegalese females
Source: BMC Med Genomics. 2022 Aug 29;15:186. doi: 10.1186/s12920-022-01337-2 (PMC9422098; doi:10.1186/s12920-022-01337-2)
Supplement: Supplementary file 6 — Additional file 6. Correspond to ELISA plates. [file 12920_2022_1337_MOESM6_ESM.pdf]

Human sCD36 data obtained by ELISA

SUNRISE; Serial number: 1312002692; Firmware: V 3.32 08/07/08; XFLUOR4 Version: V 4.51
Date: 19/7/19
Time: 16:46

Measurement mode: Absorbance
Measurement wavelength: 450 nm
Read mode: Normal

Rawdata

<>	1	2	3	4	5	6	7	8	9	10	11	12
A	3,8120	0,1940	0,1340	0,3470	0,2250	0,2300	0,4240	1,2330	1,0220	0,9950	0,9710	1,1310
B	2,3060	0,1580	0,4310	0,5380	0,2420	0,5360	2,0950	1,1080	1,0520	1,6680	0,9900	1,4960
C	1,1920	0,1560	0,3280	0,2990	0,2600	0,9920	0,8290	1,9140	1,6660	1,1800	1,0280	1,0420
D	0,8610	0,2520	0,8190	0,3990	0,9890	2,0000	1,7290	2,5070	1,5450	1,4120	1,1430	1,3550
E	0,7120	0,8610	1,7130	1,3860	1,4290	2,6580	2,2220	1,6890	1,2260	1,8200	1,2200	1,2650
F	1,4940	1,0340	1,9260	1,5570	1,7700	3,2970	2,7160	1,8700	1,3140	1,5500	1,4310	1,2680
G	2,2010	1,6120	2,9330	1,9110	2,2370	3,7460	2,5350	1,5880	0,9190	1,2120	1,2430	1,5430
H	1,1780	1,0960	2,5070	3,3320	3,6100	2,5870	2,1140	1,3050	1,1830	1,2280	1,1370	1,8860
